# Neuropilin-2 expression is inhibited by secreted Wnt antagonists and its down-regulation is associated with reduced tumor growth and metastasis in osteosarcoma

**DOI:** 10.1186/s12943-015-0359-4

**Published:** 2015-04-17

**Authors:** Tao Ji, Yi Guo, Kapjun Kim, Peter McQueen, Samia Ghaffar, Alexander Christ, Carol Lin, Ramez Eskander, Xiaolin Zi, Bang H Hoang

**Affiliations:** Department of Orthopaedic Surgery and Chao Family Comprehensive Cancer Center, University of California, Irvine, CA USA; Musculoskeletal Tumor Center, People’s Hospital, Peking University, Beijing, China; Department of Oncology, CHOC Children’s Hospital, Orange, CA USA; Department of Obstetrics and Gynecology, University of California, Irvine, CA USA; Department of Urology and Chao Family Comprehensive Cancer Center, University of California, Irvine, CA USA; Department of Orthopaedic Surgery, Montefiore Medical Center, The University Hospital for Albert Einstein College of Medicine, 3400 Bainbridge Ave, 6th Floor, Bronx, NY 10476 USA

**Keywords:** Osteosarcoma, Neuropilin, Lung metastasis, Angiogenesis, Wnt pathway

## Abstract

**Background:**

Neuropilin 2 (NRP2) isa multi-functional co-receptor to many receptors, including VEGF receptor, c-Met and others. NRP2 has recently been implicated in tumor angiogenesis, growth, and metastasis of many other cancers. However, its role in osteosarcoma remains poorly understood.

**Results:**

NRP2 was overexpressed in osteosarcoma cell lines and tissues, and associated with poor survival of osteosarcoma patients. Knockdown of NRP2 expression by short-hairpin (Sh) RNA resulted in reduced tumor growth, metastasis, and blood vessel formation of osteosarcoma. Knockdown of NRP2 expression by ShRNA also inhibited the recruitment of HUVEC cells to osteosarcoma cells. Inhibition of Wnt signaling by overexpression of secreted Wnt antagonists soluble LRP5, Frzb, and WIF1 markedly down-regulated mRNA and protein expression of NRP2 in osteosarcoma cell lines.

**Conclusions:**

Regulation of NRP2 receptor expression may represent a novel approach for treatment of osteosarcoma through retarding osteosarcoma growth, metastasis and blood vessel formation. In addition, down-regulation of NRP2 expression can be achieved by expression of secreted Wnt antagonists.

**Electronic supplementary material:**

The online version of this article (doi:10.1186/s12943-015-0359-4) contains supplementary material, which is available to authorized users.

## Background

Osteosarcoma (OS) is the most common primary malignancy of bone in children and adolescents. Despite recent advances in multimodality treatments consisting of neo-adjuvant chemotherapy and wide surgical excision, 40-50% of adolescent patients develop lung metastasis and 80% of patients with metastasis at diagnosis will relapse [1]. The survival rate has not significantly changed over past 20 years and novel therapeutic targets need to be developed.

Neuropilins, including neuropilin-1 (NRP1) and neuropilin-2 (NRP2), are multi-functional coreceptors for class 3 semaphorins (SEMA) and vascular endothelial growth factor (VEGF), essential for both neuronal guidance and cardiovascular development [2,3]. NRP1-/ mice exhibited early embryonic mortality with obvious cardiovascular defect [4]. NRP2−/− mice displayed abnormal development of peripheral lymph vessels [5]. In the vascular system, NRP1 is expressed mainly in arteries whileNRP2 is expressed in vein and lymphatic vessels [5,6]. During angiogenesis and lymphangiogenesis, NRP1 and 2 were involved in the regulation of endothelial cell properties and mediate sprouting of blood vessel and lymphatic vessels, respectively [7,8]. These findings showed the crucial role of NRP1 and 2 in the development of the vascular and lymphatic systems, respectively.

Besides being found in vascular and lymphatic channels, NRP1 and NRP2 are also expressed in a wide variety of human cancers, such as lung, breast, prostate, colon, pancreas, neuroblastoma, melanoma, etc. [3,9-14]. Oncologic studies have demonstrated that over-expression of NRP1 and NRP2 is correlated with increased vascularity, metastatic potential, advanced stage, and poor prognosis. Generally, NRP1 is predominantly found in carcinoma while NPR2 is expressed in non-epithelial tumors, such as melanoma, leukemia, neuroblastoma [15], and OS [16]. A study by Handa et al. reported that 80% of OS samples expressed NRP2 and NRP2-positive tumors showed a significant increase in vascularity, and portended a poorer prognosis [16]. However, there is still no direct evidence that blocking NRP2 in OS results in decreased blood vessel formation, tumor growth, or metastatic potential. In breast [17], colorectal [18] and pancreatic cancer [19], blocking NRP-2 resulted in suppressed tumor growth, reduced lymphangiogenesis/angiogenesis and metastasis, although the underlying mechanisms are still poorly understood.

Given the implication of NRP2 in tumor growth, angiogenesis, and metastasis, understanding the regulation of NRP2 expression is of importance. We have reported that blocking Wnt signaling by secreted antagonists resulted in a suppression of tumor growth and lung metastasis [20-24]. Angiogenesis may play a role in the regulation of tumor growth and metastasis by the Wnt signaling pathway. VEGF-A has been reported as transcriptional target gene of Wnt signaling [25]. Therefore, the effects of Wnt signaling on angiogenesis in OS and on the expression of NRP2 deserve further exploration.

In this study, we examined the expression of NRP2 in OS cell lines and tumor tissues, and correlated the expression levels with patients’ survival. By knocking down NRP2 using ShRNA, we examined the potential role of NRP2 in modulating tumor growth, invasion, metastasis and blood vessel formation in OS. In addition, we used Wnt antagonists as a means to elucidate the regulatory effect of Wnt signaling on NRP2 expression.

## Results and discussion

### NRP2 expression is correlated with metastasis and poor prognosis of OS

**Figure 1:**
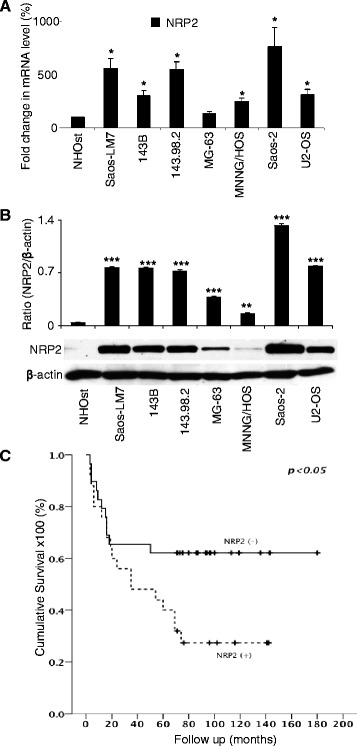
NRP2 expression is up-regulated in osteosarcoma cells. **(A)**, mRNA levels of NRP2 in normal osteoblast (NHOst) and osteosarcoma cell lines (Saos-LM7, 143B, 143.98.2, MG-63, MNNG/HOS, Saos-2, and U2-OS) were determined by real time PCR. **(B)**, the protein levels of NRP2 in NHOst and seven osteosarcoma cell lines were detected by western blot using NRP2 antibody. The relative protein levels were determined by densitometry and normalized with β-actin level. **(C)**, Kaplan-Meier survival curves of disease–specific mortality for patients whose osteosarcoma expressed or didn’t express NRP2. The log-rank test was used to compare differences between two groups. NRP2 expression was predictive of poor overall survival. Significant difference is indicated by (*) p < 0.05, (**) p < 0.01, (***) p < 0.001. Column: mean value; Error bars: SEM

Compared to normal osteoblasts (NHOst), mRNA levels of NRP2 are significantly increased in 6/7 OS cell lines and protein levels are elevated in all tested cell lines (Figure 1A & B). To examine the relationship between NRP2 expression and disease prognosis, we performed immunohistochemistry of NRP2 expression in OS patient samples using a tissue microarray. This array comprised of 24 osteoblastic, 10 chondroblastic, 19 classical, 1 fibroblastic and 1 telangiectatic OS samples. NRP2 expression was detected in 26 out of 55 samples (47.3%). Next, the cohort was dichotomized into negative and positive NRP2-staining groups and log-rank test was performed to compare survival. The results showed that expression of NRP2 correlated with poorer survival (p = 0.04) (Figure 1C). Together, these data suggest a potential role for NRP2 in the pathobiology and progression of disease in OS.

### Knockdown of NRP2 by ShRNA resulted in reduced *in vitro* and *in vivo* growth of OS

**Figure 2:**
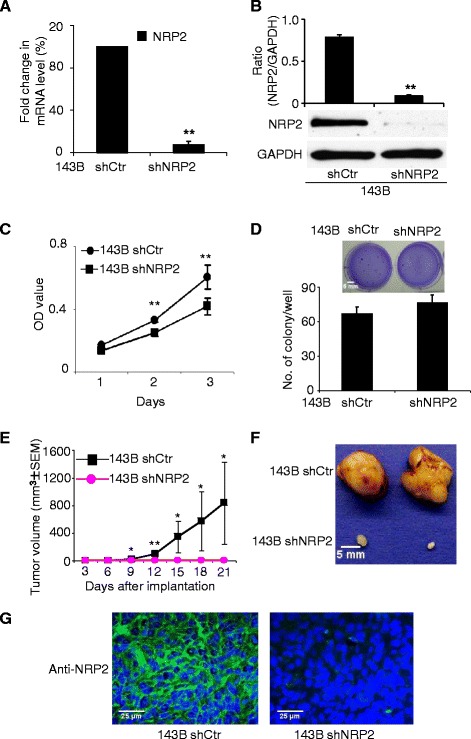
NRP2 knockdown inhibited both in vitro and in vivo tumor growth. **(A & B)**, NRP2 expression was knocked down by NRP2 shRNA in 143B cells. Knockdown efficiency was determined by real-time PCR **(A)** and Western blotting **(B)**. **(C)**, By MTT assay, NRP2 knockdown suppressed anchorage-dependent growth of osteosarcoma 143B cells. **(D)** Soft agar assay. NRP2 knockdown did not reduce the number of colony formed by 143B cells. 143B cells transfected with ShNRP2 formed smaller colony than control vector transfected cells as shown in the representative images of soft agar (insert). **(E) & (F)** NRP2 knockdown inhibited the in vivo tumor growth in xenograft nude mice model. 1 × 10^6^143B cells were inoculated in NCR nu-nu nude mice. Tumor size was measured every 3 days and a tumor growth curve was created **(E)**. Points, mean tumor volume; Bars, SEM. **(F)**, Representative picture of tumor harvested at day 21. **(G)**, the knockdown of NRP2 expression in tumor samples was confirmed by immunofluorescence staining using NRP2 antibody. Significant differences are indicated by: (*) p < 0.05, (**) p < 0.01. Column: mean value; Error bars: SEM.

Given the high endogenous level of NRP2 in OS cells, we performed NRP2 knockdown by ShRNA to examine its effects on the growth of OS cells. NRP2 mRNA (Figure 2A) and protein levels (Figure 2B) were both efficiently knocked down by ShNRP2, while NRP1 expression level remained intact (Additional file 1: Figure S1A), suggesting that NRP2 knockdown was specific. NRP2 knockdown inhibited the growth of 143B cells by 23.9% (p < 0.01) at day 2 and by 30.8% (p < 0.01) at day 3 compared to control 143B cells treated with a non-targeting ShRNA (Figure 2C). A similar growth inhibition by ShNRP2 was also found in Saos-2 cells (Additional file 1: Figure S1B). We then performed soft agar assays to examine anchorage-independent growth of tumor cells. NRP2 knockdown did not reduce the number of colony formed by 143B and Saos-2 cells in soft agar (Figure 2D and Additional file 1: Figure S1C). However, the colony size was reduced (Figure 2D insert and Additional file 1: Figure S1C insert), suggesting that ShNRP2 preferentially inhibited tumor growth instead of tumorigenesis. Flow cytometry revealed a mild increase in the number of apoptotic cells following NRP2 knockdown (Additional file 1: Figure S1D). We then examined the effect of NRP2 down-regulation on *in vivo* tumor growth using a xenograft model. NRP2 knockdown in xenograft tumor samples was confirmed by immunofluorescence (Figure 2G). As shown in Figure 2E&F, NRP2 knockdown in OS cells has a significant inhibitory effect on tumor growth. Compared to the ShRNA control group, a knockdown of NRP2 reduced tumor growth by 95.3% at day 9 (P < 0.05), 99.1% at day 12 (P < 0.01), 98% at day 15 (P < 0.05), 97.9% at day 18 (P < 0.05), and 99.2% at day 21 (P < 0.05). Interestingly, the *in vivo* inhibition of tumor growth by ShNRP2 is not proportional to its *in vitro* anti-proliferative effect, suggesting that non-proliferative mechanisms may mediate the *in vivo* effects.

### NRP2 knockdown resulted in reduced tumor invasion, migration, cell-cell adhesion, and lung metastasis *in vivo*

**Figure 3:**
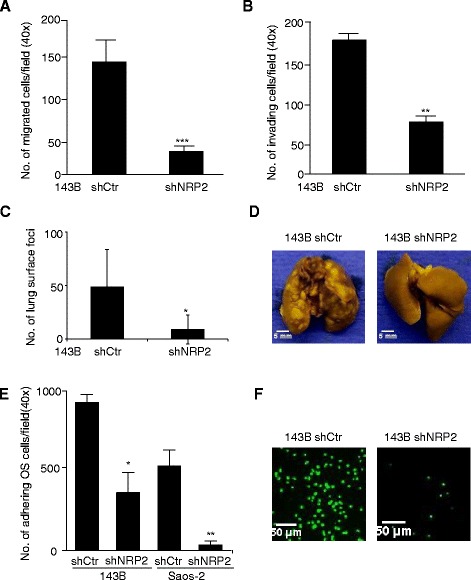
Knockdown of NRP2 inhibited the tumor invasion, migration and lung metastasis of osteosarcoma. **(A)**, Migration assay. The BD chamber system without Matrigel coating was used to evaluate the migration of shNRP2 and control vector transfected osteosarcoma 143B cells. The migration of 143B cells was significantly inhibited by NRP2 knockdown. **(B)**, Matrigel invasion assay was performed in BD chamber system coated with Matrigel, using shNRP2 and control vector transfected osteosarcoma 143B cells. There were less NRP2 depleted 143B cells invaded through the matrigel coated porous membrane. **(C)**, Knockdown of NRP2 in 143B cells reduced the lung metastasis of osteosarcoma in an orthotopic lung metastasis mouse model. Mouse lungs were fixed in Bouin’s solution, and the number of lung surface metastatic nodules was counted and graphed. Each group contained 10 mice and the experiment was repeated 3 times **(D)**, Representative photographs of lungs with metastatic nodules of osteosarcoma. **(E)**, NRP2 knockdown significantly reduced both 143B and Saos-2 cells adherence to the endothelial monolayer. The mean cell number was calculated from 10 fields (×100). **(F)**, Representative images of GFP transfected tumor cells adhering to the endothelial monolayer. Significant differences are indicated by: (*) p < 0.05, (**) p < 0.01, (***) p < 0.001. Column: mean value; Error bars: SEM.

To examine the effects of NPR2 knockdown on OS cell migration and invasion, we performed transwell motility and Matrigel invasion assays. Both migration and invasion were significantly inhibited by NRP2 knockdown in 143B and Saos-2 cells (Figure 3A&B and Additional file 1: Figure S2A,B). The levels of phosphorylated Akt and MAPK were significantly decreased after NRP2 knockdown (Additional file 1: Figure S2C). When tested in an orthotopic lung metastasis xenograft model, NRP2 knockdown in 143B cells led to a significant decrease in lung metastatic nodules (Figure 3C&D). Interactions of tumor cells with endothelial cells are known to mediate metastasis. NRP2 has been reported to act as an adhesion molecule [26], we speculated that the interaction of OS cells and endothelial cells may promote the homing of OS cells to the lung endothelium. Using an *in vitro* cell adhesion model, NRP2 knockdown significantly impaired the adhesion of 143B and Saos-2 cells to the endothelial monolayer (Figure 3E&F).

### NRP2 knockdown is associated with decreased blood vessel density in OS

**Figure 4:**
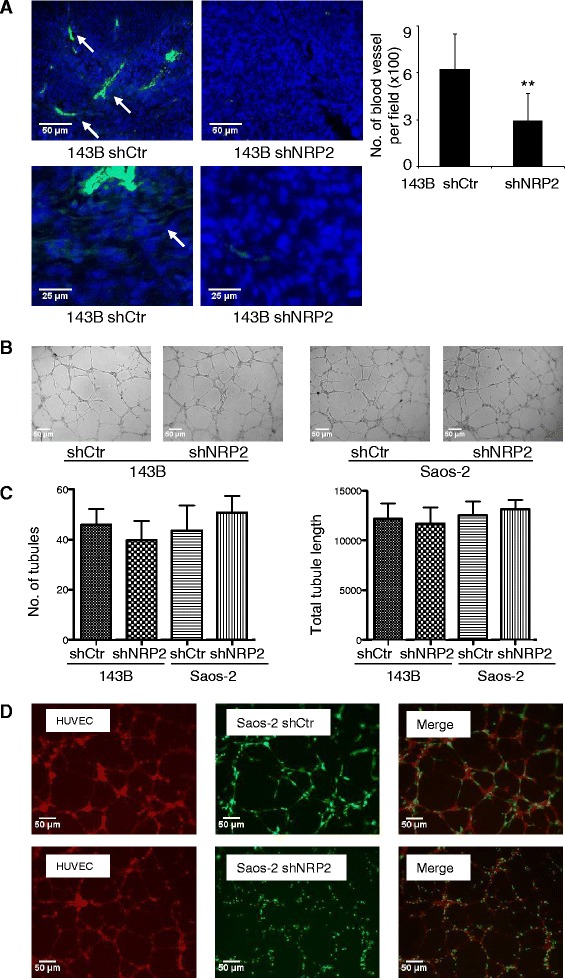
Knockdown of NRP2 resulted in decreased blood vessel density of OS *in vivo*. **(A)**, Blood vessels (top panel) and capillaries (bottom panel) in tumor samples were visualized by immunohistochemistry with CD31 antibody. Tumor cell nuclear was stained with DAPI. Number of blood vessels per field (100×) was calculated and graphed (Right). Column, mean number of blood vessel per field (x100); Error bars, SEM. **(B & C)**, Matrigel tube formation assay: no difference was found in the number of tubules formed by HUVEC in conditioned medium from shNRP2 OS cells and shRNA control cells. **(B)** Representative photographs of tubules formed by HUVEC cells on Matrigel. **(C)** The tubular number was calculated and graphed, Column: mean number of tubules; Error bars: SEM. **(D)**, tumor-endothelial co-culture tube formation assay: The HUVEC cells were stained with CellTracker Red CMTPX dye and the tumor cells were transfected with shNRP2 vector with GFP expression. Left panel: tubules formed by HUVEC (red); middle panel: tumor cells (green) attached on tubules; right panel: merged image (right) of HEVEC tubules (red) and attached tumor cells (green). NRP2 depleted tumor cells sustained distinct morphologic changes compared to control cells (bottom panel).

Given the hypervascular nature of OS tumors and the role of NPR2 in angiogenesis, we hypothesize that knockdown of NRP2 expression may exert a negative effect on angiogenesis in OS. Using an athymic nude mouse model, we examined tumor blood vessels and capillaries in NRP2 knockdown and control tumors by anti-mouse CD31 immunostaining. Figure 4A showed that knockdown of NRP2 resulted in significantly decreased blood vessel density in NRP2 knockdown tumor (p < 0.01). Interestingly, capillaries in NRP2 knockdown tumors were rarely observed compared to those in control tumors (Figure 4A). However, in an *in vitro* model, the conditioned medium from NRP2 knockdown OS cells did not suppress HUVEC tube formation as shown by automated quantification of number of tubules and total tubule length (Figure 4B&C). No significant difference was observed in the levels of VEGF-A in the conditioned medium from control OS cells and NRP2 knockdown cells (Additional file 1: Figure S3). In addition, in a co-culture model, the close contact between HUVEC and OS tumor cells did not significantly affect the capacity for tube formation (Figure 4D). However, during co-culture, NRP2 depleted OS cells underwent distinct morphologic change with most of the tumor cells became round and distributed along the endothelial tubes (Figure 4D). The significance of this change in morphology and the underlying mechanisms remain to be determined.

### NRP2 knockdown inhibited endothelial recruitment by OS cells

**Figure 5:**
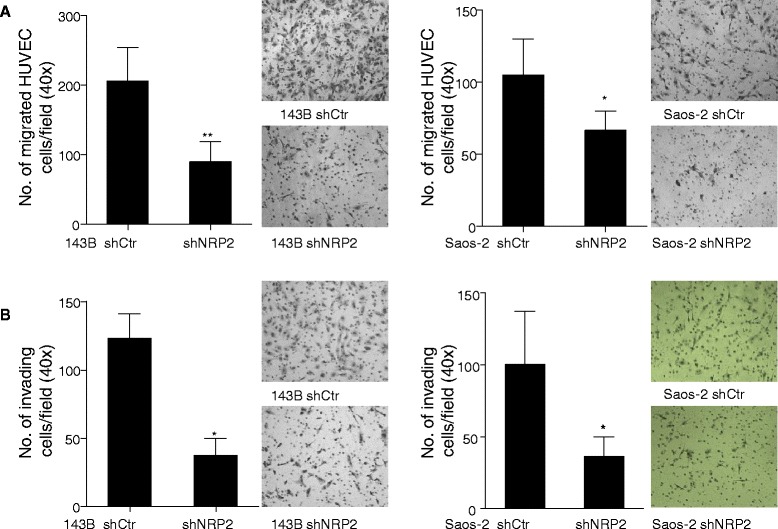
NRP2 knockdown suppressed endothelial recruitment by osteosarcoma cells. **(A)**, in a trans-well co-culture migration model, less endothelial cells migrated through the porous insert membrane of the Chamber in shNRP2 group compared to control group. shNRP2 depleted osteosarcoma cells vs control cells were seeded in the lower chamber. HUVECs were seeded in the top insert chamber. Insert picture: Representative photograph of migrated HUVECs. **(B)**, in a trans-well co-culture invasion model, less endothelial cells invaded through the matrigel coated porous insert membrane of the Chamber in shNRP2 group compared to control group. shNRP2 depleted osteosarcoma cells vs control cells were seeded in the lower chamber. HUVECs were seeded in the top insert chamber. Insert picture: Representative photograph of invaded HUVECs. Significant differences are indicated by: (*) p < 0.05, (**) p < 0.01. Column: mean migrated/invaded cells per field (40×); Error bars: SEM.

As seen in Figure 4, conditioned medium from tumor cells with NRP2 suppression did not significantly affect endothelial tube formation *in vitro*. However, ShNRP2 transfection negatively affected blood vessel formation in the *in vivo* mouse model. We speculated that NRP2 knockdown in tumor cells may suppress the recruitment of endothelial cells. To test this, we performed a trans-well co-culture model in which NRP2 knockdown OS cells were placed in the bottom well and HUVECs were placed in the upper porous trans-well insert. We observed that less HUVECs in NRP2 knockdown group migrated through the porous insert than those in the control group (Figure 5A&B). In addition, decreased recruitment of HUVEC was observed in both Matrigel coated and uncoated inserts, suggesting that migration and invasion of endothelial cells were inhibited by NRP2 knockdown in tumor cells (Figure 5A&B). These findings suggest that inhibition of vessel formation in ShNRP2 tumors may be mediated by decreased endothelial recruitment.

### NRP2 expression is regulated by canonical Wnt signaling

**Figure 6:**
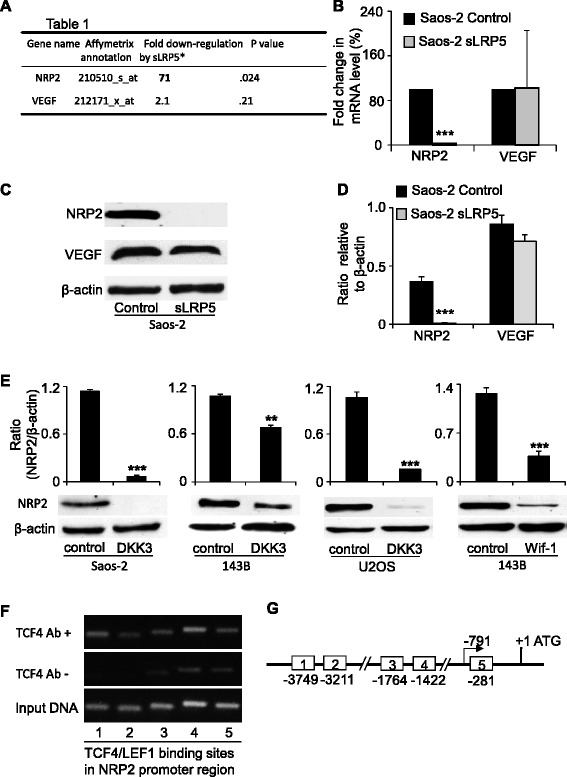
NRP2 expression is regulated by Wnt signaling pathway. **A**, Genechip® microarray showed the down-regulated expression of NRP2 in Wnt antagonist sLRP5 transfected osteosarcoma Saos-2 cells. VEGF expression is intact. Real time PCR **(B)** and western blot **(C)** with accompanying densitometric assay **(D)** confirmed the down-regulated mRNA and protein level of NRP2 in Wnt antagonist sLRP5 transfected Saos-2 cells, while VEGF level remains intact. **E**, western blot and accompanying densitometry showed the down-regulated NRP2 expression in additional Wnt antagonist DKK3 transfected Saos-2 cells, 143B cells and U2-OS cells, and Wif-1 transfected 143B cells. **F**, Chromatin immunoprecipitation (ChIP) assay verified the binding of TCF4 on the five binding sites in NRP2 promoter region. **G**, schematic representation of the binding sites of TCF/LEF on the 3-kb promoter region of NRP2 genes.

Genechip® microarrays showed that blocking canonical Wnt signaling by a soluble LRP5 receptor (sLRP5) down-regulated NRP2 > 70 fold (Figure 6A), while VEGF expression level remain unchanged, as confirmed by real-time PCR (Figure 6B) and western blot (Figure 6C & D). These results suggest that NRP2 is negatively regulated by secreted Wnt antagonists. Over-expression of the Wnt antagonist DKK3 or Wif-1 in Saos-2, 143B, U2OS cells resulted in reduced expression of NRP2 (Figure 6E). These findings implicate NRP2 as a potential Wnt target gene in OS. By using Genebank and MatInspector software, five putative TCF4 binding sites were identified in the NRP-2 promoter (Figure 6G). Chromatin immunoprecipitation (ChIP) assays were performed to determine whether these sites are capable of binding TCF. An anti-TCF4 antibody was used to immunoprecipitate DNA fragments that can bind TCF4. These fragments were then amplified using primers spanning each of the five TCF binding sites. As shown in Figure 6F, TCF4 bound all five binding sites in NRP2 promoter region, suggesting that NRP2 is a potential transcriptional target of canonical Wnt signaling.

### Discussion

In the present study, we show that NRP2 is significantly elevated in OS cell lines compared to normal osteoblasts. Furthermore, the expression of NRP2 correlates with a poor prognosis of OS patients. To the best of our knowledge, this study also represents the first line of evidence that transcriptional regulation of neuropilin involves the Wnt signaling pathway. Our *in vitro* data suggest that NRP2 modulates the recruitment of endothelial cells as a means to enhance tumor angiogenesis. Our *in vivo* experiments corroborate these data by demonstrating that inhibiting NRP2 by ShRNA impairs blood vessel formation, tumor growth, and metastatic potential of OS. These data suggest that NRP2 serves a tumor promoting function in OS. Therefore, suppression of NRP2 may be a novel strategy for the treatment of OS metastasis and progression.

Knockdown of NRP2 by ShRNA only resulted in a moderate inhibition of *in vitro* cellular growth and a mild apoptotic effect in OS. However, NRP2 knockdown significantly abrogated the *in vivo* growth of OS tumors in a mouse model. These findings indicate that the effects of NRP2 knockdown *in vivo* is not a direct result of inhibiting tumor cell proliferation. Interestingly, our study showed that tumor samples from the ShNRP2 group displayed lower blood vessel density, suggesting NRP2 enhances blood vessel formation in OS. This finding is consistent with previous reports in breast [17], colorectal [18], and pancreatic cancer [19]. However, none of those reports investigated the interaction of NRP2 suppressed tumor cells and endothelial cells. In this study, we used tumor cell-HUVEC co-culture model to examine the effect of NRP2 knockdown in tumor cells on HUVEC tube formation and recruitment. Unexpectedly, knockdown of NRP2 in OS cells preferentially inhibit the recruitment of HUVEC rather than inhibiting the capacity of HUVEC to form endothelial tubes. To the best of our knowledge, NRP2-mediated endothelial recruitment by OS cells represents a novel finding in this study as this phenomenon has not been well described. Vascular endothelial cells recruitment plays a pivotal role in vessel sprouting and angiogenesis [27]. Blocking recruitment of endothelial progenitor cells by anti-angiogenic drugs has been associated with a reduction in tumor size and blood flow [28]. Molecular mechanisms underlying endothelial cell recruitment by NRP2 in tumor cells remains to be explored.

VEGF, SDF-1 [29], and CXC chemokine [30] have been reported to mediate angiogenesis including endothelial cell recruitmeng [29]. In the present study, our data indicated that NRP2 knockdown did not affect the secretion of VEGF from tumor cells. A series of other soluble factors deserve further exploration. In addition, the recruitment of angiocompetent BMDCs (bone-marrow-derived dendritic cells), TEMs (Tie2-expressing monocyte), TAMs (Tumor-associated macrophages), neutrophils, mast cells and CD11b + GR-1 + myeloid-derived suppressor cells, which release angiogenic factors such as VEGF, and MMP [31] may also play an important role in endothelial recruitment and angiogenesis in tumor. The recruitment of angiocompetent immune cells by NRP2 depleted tumor cells deserves further study as well.

Tumor-endothelial communication has mutual influence on both sides. Our data showed that NRP2 knockdown in tumor cells not only changed the behavior of tumor cells but also altered the interaction between OS and endothelial cells. Interestingly, during co-culture with endothelial cells, the NRP2-suppressed OS cells developed significant morphological change. The significance and mechanism underlying the morphological change of Sh-NRP2 cells remains to be explored. Our previous study demonstrated decreased metastatic capacity of morphologically changed OS cells by Wnt antagonists [22,32]. Further experiments are needed to determine if the co-culture and NRP2 knockdown induced OS cell morphology change is one of the mechanisms that resulted in the decreased lung metastasis in OS.

Pulmonary metastasis represents a poor prognostic factor that still remains as a therapeutic bottleneck in OS. NRP2 expression has been shown to correlate with aggressiveness and metastasis of several types of malignancy [3]. In this study, our tissue microarray data clearly shows that NRP2 expression correlates with a worse prognosis, strongly implicating its role in OS progression and metastasis. NRP2 knockdown in OS cells exhibited reduced motility and invasion, suggesting a direct effect of NRP2 on metastatic capacity of OS cells. Others have shown that NRP2 promotes extravasation and metastasis in renal cell carcinoma by interaction with endothelial α5 integrin [26]. Here, our *in vivo* studies demonstrated that NRP2 knockdown significantly inhibited pulmonary metastasis of OS in a xenograft mouse model. Cancer cell adhesion involves interacting with adjacent cancer cells as well as endothelial cells and extracellular matrix. NRP1 was initially identified as a cell-cell adhesion molecule [33]. Therefore, it is of interest to determine whether NRP2 is involved in cancer to endothelial cell adhesion. Goel and colleagues reported that NRP2 regulated α6β1 integrin and enabled the formation of focal adhesion of breast cancer [34]. Cao et al. used atomic force microscopy to precisely measure the attachment and detachment of cancer cells to endothelial cells via NRP2 binding to α5 integrin [26]. Using endothelial adhesion assays, we demonstrated that NRP2 is required for OS cell adhesion to endothelial cells, consistent with recent reports in the literature. Clearly, findings from this study and others have deepened our understanding of the important role of NRP2 in tumor metastasis, indicating its potential as a novel therapeutic target.

VEGF-A has been reported as a transcriptional target of Wnt signaling in colon cancer [25]. Unlike NRP2, we found that VEGF-A expression was not suppressed in OS cells by the Wnt antagonist sLRP5. Due to diverse functions of Wnt antagonists, we also sought to use other secreted antagonists to examine the consequence of Wnt blockade on NRP2. Blocking Wnt signaling by additional antagonists such as Wif-1 and DKK3 led to a similar suppression of NRP2, strongly implicating this receptor as a downstream Wnt target. Furthermore, chromatin IP assays confirmed the existence of TCF-binding sequences within 3kbp upstream of the transcription start site for the NRP2 gene. These regions presumably function as transcriptional regulatory elements. Whether the NRP2 promoter responds to the β-catenin/TCF complex still remained undetermined. However, these findings suggest a novel role for Wnt signaling as a modulator of tumor angiogenesis by regulating NRP2.

In this study, we found that blocking Wnt signaling by DKK3 resulted in variable inhibitory effects on NRP2 in different OS cell lines. The reason for this variation may be related to the ability of Dkk-3 to inhibit Wnt signaling being cellular context-dependent. We previously demonstrated Wnt inhibition by DKK3 in OS Saos-2 cells as evidenced by antagonism of cytoplasmic β-catenin [22]. However, ectopic Dkk-3 expression in prostate cancer cells activates c-Jun NH2-terminal kinase (JNK) leading to apoptosis but did not affect β-catenin level, suggesting a role for Dkk-3 in the Wnt/PCP rather than in the Wnt/β-catenin pathway [35]. Despite this variation, secreted Wnt antagonists appear to down-regulated NRP2 expression in OS cells, suggesting that Wnt signaling modulates tumor angiogenesis via NRP2.

## Conclusion

We demonstrated that inhibition of NRP2 expression by shRNA knockdown impaired tumor growth, invasion, and blood vessel formation in OS. NRP2 modulates tumor blood vessel formation by altering the recruitment of endothelial cells to OS tumor cells. In addition, NRP2 can be down-regulated by overexpression of secreted Wnt antagonists such as sLRP5, Frzb, and WIF1. Our study also suggests that NRP2 expression is transcriptionally regulated by canonical Wnt signaling.

## Methods

### Cell lines and plasmids

Normal Human Osteoblasts (NHOST) were obtained from Cambrex Bio Science and maintained in Osteoblast Growth Media (Lonza). Saos-LM7 was a gift from Dr. Eugenie Kleinerman (MD Anderson Cancer Center, Houston, TX). Human OS cell lines 143B, 143.89.2, Saos-2, MNNG/HOS and U2OS (American Type Culture Collection), Saos-LM7, and OS160 were maintained in MEMα medium supplemented with 10% fetal bovine serum (FBS), supplemented with penicillin/streptomycin. Human Umbilical Vein Endothelial Cells (HUVEC) were from ATCC (American Type Culture Collection, Manassas VA) and maintained in EGM-2 medium supplemented with 2% FBS and VEGF. All cells were cultured at 37°C in a humidified incubator with 5% CO2. PCDNA3.1 Directional TOPO Expression vector was obtained from Invitrogen. An Ultimate ORF WIF-1 clone (ID: IOH11153) was obtained from Invitrogen and subcloned into the PCDNA3.1 TOPO vector. The dominant-negative, soluble LRP5 plasmid (sLRP5, a generous gift of Matthew Warman, Case Western Reserve University, Cleveland, OH), was constructed by deleting the transmembrane and cytoplasmic domains of LRP5, resulting in a secreted protein that binds and prevent Wnt ligands from activating the native LRP5 receptor.

### Tissue microarrays (TMA) and survival analysis

Patient OS tissue microarrays (TMA) were purchased from IMGENEX. NRP-2 staining was conducted by using rabbit anti-NRP2 antibody and anti-rabbit immunostaining kit from R&D system. NRP2 staining intensity of the tumor tissue was scored as no staining (−), weak staining (+), moderate staining (++), and strong staining (+++). The clinical information of tissue microarray samples included follow-up duration and survival status. Kaplan-Meier survival curve was created to represent disease–specific mortality for patients with or without NRP2 expression. The log-rank test was used to compare differences between two groups.

### RNA interference

For NRP2 knockdown experiment, OS cell lines were transfected with Lipofectamine 2000 (Life Technologies) with pGFP-V-RS HuSH shRNA vectors (Origene) containing either the scrambled shRNA cassette TR30013 (control) or human NRP2 targeting shRNAs. 143B or Saos-2 cells constitutively expressing the puromycin resistance gene and human NRP2 shRNA were selected and expanded in media containing 1 μg/mL puromycin. The pooled stable transfectants (to avoid cloning artifacts) were examined.

### RNA isolation and real time quantitative RT-PCR

Total RNA was isolated using TRIzol reagent (Invitrogen) according to the manufacturer’s instructions. Reverse transcription of total RNA was performed using the Reverse Transcription System kit (Promega, Madison, WI) with random primers. Real-time RT-PCR was performed as previously described [21]. PCR condition was as follows: 45 cycles of 30 seconds at 95°C, 30 seconds at 58°C, 60 seconds at 72°C. Relative fold change in mRNA expression compared to control was calculated using the comparative Ct method. Ct is the cycle number at which fluorescence intensity first exceeds the threshold level. ∆ Ct is Ct (target gene) -Ct (actin). Gene-specific primer sequences are available upon request. Specificity of amplification products were verified by agarose gel electrophoresis and melting curve analysis [24].

### Western blot

Twenty to 80 μg of protein lysate was separated electrophoretically on denaturing SDS-polyacrylamide gel, transferred to nitrocellulose membranes, and probed with primary antibodies. Blots were exposed to horseradish peroxidase-conjugated secondary antibodies and visualized by an ECL detection system according to the manufacturer’s protocol (Amersham Biosciences, Piscataway, NJ). For loading control, the initial western blot was placed in Restore Western stripping buffer (Thermo Scientific) for 15 min to remove the antibody (primary and secondary), washed in water for 5 min, blocked with 5% milk for 1 hour, and probed with β-actin antibody (Santa Cruz Biotechnology, Santa Cruz, CA; Catalog # sc-130301) [20,24]. The primary antibodies used in western blot included NRP2, GAPDH (Santa Cruz Biotechnology, Santa Cruz, CA. NPR2 catalog # sc-13117; GAPDH catalog # sc-137179); VEGF, DKK3 (Abcam, Cambridge, MA. VEGF catlog # ab46154; DKK3 catalog # ab136101); Wif-1 (Cell Signaling Technology, Danvers, MA, Catalog # 5652); Each western blot was repeated 3 times. Blots were quantitated by densitometry using Image J (Software, NIH, Bethesda, MD, USA) and normalized to a housekeeper marker β-actin or GAPDH [36]. The intensity of each tested marker is presented as a ratio of a tested mark/β-actin or GAPDH.

### Immunofluorescence staining

Tumor and lung tissues were fixed in formalin and sectioned. Antigen retrieval was done using 10 mmol/L sodium citrate (pH 6.0) at 95°C for 15 min. After blocking with PBS containing 3% bovine serum albumin for 1 h, cells were incubated with an NRP2 antibody (Santa Cruz Biotechnology, Santa Cruz, CA, Catalog# sc-13117) for 12 h. After washing, cells were incubated with an Alexa-488 conjugated secondary antibody (Molecular Probes, Eugene, OR, Catalog # A-11059) and mounted in Vectashield with 4’, 6-diamidino-2-phenylindole (DAPI, Vector Laboratories, Burlingame, CA). Localization of immunostaining was analyzed by laser scanning confocal microscopy (X40 magnification) using the 488-excitation wavelength [32].

### Flow cytometry

For apoptosis detection, cells were stained with FITC-labeled annexin V and propidium iodide (PI) (BD Biosciences, San Jose, CA). After incubations, floating cells were first harvested and adherent cells were trypsinized and pooled together. Cells were washed twice with cold PBS and resuspended in 1× binding buffer at a concentration of 1 × 10^6^ cell/ml. Transfer 100ul of the solution (1 × 10^5^ cells) to a 5 ml culture tube. Add 5ul of FITC Annexin V and 5ul PI. Gently vortex the cells and incubate for 15 min at room temperature in the dark. After the incubation period, 400ul of 1 × binding buffer was added to each tube. The stained cells were analyzed by flow cytometry in one hour. Each flow-cytometry experiment was repeated three times [37].

### Blood vessel density assay

Immunofluorescence was used to determine blood vessel density with CD31 (Abcam), a marker for vascular endothelium, and the blood vessel staining was analyzed with a Nikon Eclipse TE2000-S fluorescent microscope (magnification, 100X) using the 488-excitation wavelength. The vessel density was determined by counting the number of blood vessels in 10 fields (magnification, 100X) of slides from each group and the result was recorded as mean number of blood vessels per field.

### Motility and invasion assay

Motility and invasion assays were performed using 24-well invasion chamber system (BD Biosciences, MA) as previously described [21]. The matrigel coated inserts were used for invasion assay. The uncoated inserts were used for motility assay. 1 × 10^5^ cells for invasion assay and 5 × 10^4^ cells for motility assay were seeded in the upper chamber in serum-free MEMα medium. MEMα medium with 10% FBS (used as a chemoattractant) was placed in the bottom well. Incubation was carried out for 48 h for invasion assay or 24 h for migration assay, at 37°C in humidified air with 5% CO2. Non-invasive or non-migrating cells in the upper chamber were then removed with a cotton swab. Invading or migrating cells on the bottom of the inserts were fixed with methanol and stained with hematoxylin. The number of invading or migrating cells was determined by counting five fields (100×) under the microscope, and calculated as mean number of cells per field. All experiments were performed in triplicate.

### Endothelial recruitment

Twenty thousand tumor cells were seeded into 24-well plates approximately 24 h before the start of the recruitment assay. HUVEC cells were serum starved in EGM-2 medium supplemented with 0.2% FBS for 24 h. HUVEC cells were then suspended at 4 × 10^4^ HUVECs per ml of 0.2% FBS EGM-2. 500 μl of the suspension was then added into each insert and the recruitment assay was allowed to proceed for 12–16 h depending on tumor cell lines. After completion of the assay, the inserts were fixed with methanol for 5 min and stained with hematoxylin. The number of migrating or invading cells was determined by counting 5 fields at × 100 magnification.

### Endothelial adhesion assay

HUVEC cells were cultured in 6-well plate and allowed to grow to confluence. 2 × 10^4^ tumor cells were added into the wells pre-cultured HUVEC cells for 30 minutes at 37°C. The wells were washed with PBS gently to remove non-adherent cells. Five images were taken. The number of tumor cells adhering to the HUVEC cell was then quantified using ImageJ (NIH).

### Soft agar colony formation assay

Soft agar colony formation assay was performed using six-well plates as previously described [24]. Each well contained 2 mL of 0.8% agar in complete medium as the bottom layer, 1 ml of 0.35% agar in complete medium and 6000 cells as the feeder layer, and 1 ml complete medium as the top layer. Cultures were maintained under standard culture conditions. The number of colony was determined with an inverted phase-contrast microscope at × 100 magnification. A group of >10 cells was counted as a colony. The data are shown as mean number of colony ± SEM of four independent wells at 20 days after the start of cell seeding [24].

### In vivo tumorigenesis model

4-week old male nu/nu nude mice (Taconic) were purchased and housed in pathogen-free conditions for additional 4 weeks before tumor inoculation. The animal protocol was approved by the Institutional Animal Care Utilization Committee (IACUC). Sh-NRP2 transfected and vector control transfected 143B cells were grown to 80% confluence, and re-suspended in PBS and injected subcutaneously (S.C.) into the flank of 8-week old nude mice at 1 × 10^6^ cells/0.1 ml. Tumor size was measured every 3 days using a caliper. The tumor volume was calculated by the formula 1/6 πab^2^ (π =3.14, a = long axis and b = short axis of the tumor). Growth curves were plotted from the mean tumor volume ± SEM from 10 animals in each group. 21 days after injection, the animals were sacrificed and tumors were harvested, measured, weighed, and fixed in 10% formalin [20,24]. Each experiment was repeated 3 times.

### In vivo lung metastasis model

4-week old male nu/nu nude mice (Taconic) were were purchased and housed in pathogen-free conditions for additional 4 weeks before tumor inoculation. shNRP2 transfected and vector control transfected 143B cells were grown to 80% confluence, and re-suspended in PBS. 0.03 ml of cell suspension (1 × 10^7^ cells/ml) was injected percutaneously into the tibia of anesthetized 8-week old nude mice. After 4 weeks, the animals were sacrificed according to an IACUC-approved protocol. Lungs were harvested, fixed in Bouin’s solution, and the number of lung surface metastatic nodules was counted and graphed. To determine the number of lung surface metastatic nodules per field, we count 10 fields (×100) to calculate the mean value for each group. Mean number of lung nodules was compared between shNRP2 transfected and vector control transfected groups. Each group contained 10 mice and each experiment was repeated 3 times. Microscopic lung metastases were visualized on 5-μm paraffin embedded sections stained with H & E [20].

### HUVEC Tube Formation Assay

In order to examine the effect of NRP2 knockdown on angiogenesis, matrigel tube formation assay was performed using Human Umbilical Vein Endothelial Cells (American Type Culture Collection, Manassas VA). Tube formation assay was performed according to the manufacturer’s protocol (BD, Endothelial Cell Tube Formation Assay). Briefly, HUVEC cells were serum starved in EGM-2 media supplemented with 0.2% FBS for 24 h. 96-well culture plates were coated with 50 μl Matrigel (BD Biosciences, San Hose, CA), that was allowed to solidify for 1 hour at 37°C. The pre-treated HUVEC cells were then seeded in the well at 3 × 10^4^ cells/well. Meanwhile, the conditioned medium was collected and added in each wells. Then the HUVEC cells were incubated at 37°C for 6 hours. For tumor cell-HUVEC co-culture model, HUVEC cells were labelled with CellTracker Red CMTPX dye (Invitrogen) for 30 min and subsequently treated in EGM-2 media supplemented with 2% FBS for 30 min. The tumor cells and HUVEC cells were trypsinized and re-suspended at 4 × 10^4^/ml and 2 × 10^5^/ml respectively in EGM-2 media supplemented with 2% FBS. The tumor and HUVEC cell suspensions were then mixed at a 1:1 ratio and 100 μl of each mixture was seeded into each well of the tube formation assay plate. The assay plate was incubated at 37°C for 12 h. Images of each well were taken and processed using Angiogenesis Analyzer for ImageJ software (NIH) to obtain the number of tubules and total tubule length per image.

### Chromatin immunoprecipitation (ChIP) analysis

By analysis of promoters using Genebank and MatInspector software, five TCF/LEF binding sites in NRP2 promoter region were found. In order to verify the binding of TCF/LEF on NRP2 promoter region, Chromatin immunoprecipitation (CHIP) analysis was performed using Upstate Chromatin immunoprecipitation (CHIP) Assay Kit (Millipore, San Diego, CA). Briefly, 1x10^6^ 143B cells were seeded in 10 cm dish. After overnight incubation, 1% formaldehyde was directly added to the culture medium and incubated for 10 minutes at 37°C to cross link histones to DNA. Cells were then lysed and sonicated to shear DNA to between 200–1000 base pairs. TCF4 antibody (3 μg/mL) was added and incubated overnight with rotation. 60 μL Protein A Agarose was added to precipitate the DNA/antibody complex. After sequentially washing with low salt immune complex wash buffer, high salt immune complex wash buffer, LiCl immune complex wash buffer, and TE buffer, DNA/antibody complex was eluted from antibody. After histone-DNA crosslink at 65°C for 4 h, DNA was recovered by phenol/chloroform extraction and ethanol precipitation. Polymerase chain reaction (PCR) was performed to amplify the TCF/LEF binding sites. Five pairs of primer for NRP2 promoter, which span each TCF/LEF binding site, were used. The primer sequences are available upon request.

### Statistical analysis

Comparisons of number of colony, fold change in levels of mRNA, and the blood vessel density between different transfection groups were conducted using Student’s t-test. For tumor growth experiments, repeated measures ANOVA was used to examine the differences in tumor volume among different transfection groups at each time points. Additional post-test was done to examine the differences in tumor volume between vector control and Sh-NRP2 transfection groups at each time point by the conservative Bonferroni method. All statistical tests were two sided. P < 0.05 was considered statistically significant.

## Additional file

Additional file 1: Figure S1. NRP2 knockdown inhibited in vitro tumor growths. **(A)**, NRP2 expression was specifically knocked down by NRP2 shRNA in 143B and Saos-2 cells, while NRP1 level was intact. Knockdown efficiency was determined by Western blotting and quantified by densitometric assay. **(B)**, By MTT assay, NRP2 knockdown suppressed anchorage-dependent growth of Saos-2 cells. **(C)**, Soft agar assay. NRP2 knockdown didn’t significantly reduce the number of colony formed by Saos-2 cells, but decreased the size of colony as shown in the representative images of soft agar (insert). **(D)** There were slightly higher percentage of early and late apoptosis in shNRP2 OS cells, compared to control cells. **Figure S2.** NRP2 depletion significantly suppressed migration and invasion of Saos-2 cells. (A), Migration assay. The BD chamber system without Matrigel coating was used to evaluate the migration of osteosarcoma Saos-2 cells transfected with shNRP2 and control vector. **(B)**, Matrigel invasion assay was performed in BD chamber system coated with Matrigel, using shNRP2 and control vector transfected osteosarcoma Saos-2 cells. **(C)**, Western blot and accompanying densitometry demonstrated the inhibition of AKT and MAPK phosphorylation by NRP2 knockdown in 143B cells. **Figure S3.** ELISA demonstrated no difference of VEGF-A level in the condition medium from shNRP2 and control vector transfected 143B cells.
