# Supplementary figures and images for: Neuropilin-2 expression is inhibited by secreted Wnt antagonists and its down-regulation is associated with reduced tumor growth and metastasis in osteosarcoma
Source: Mol Cancer. 2015 Apr 17;14:86. doi: 10.1186/s12943-015-0359-4 (PMC4411772; doi:10.1186/s12943-015-0359-4)

Suppl. Fig.1

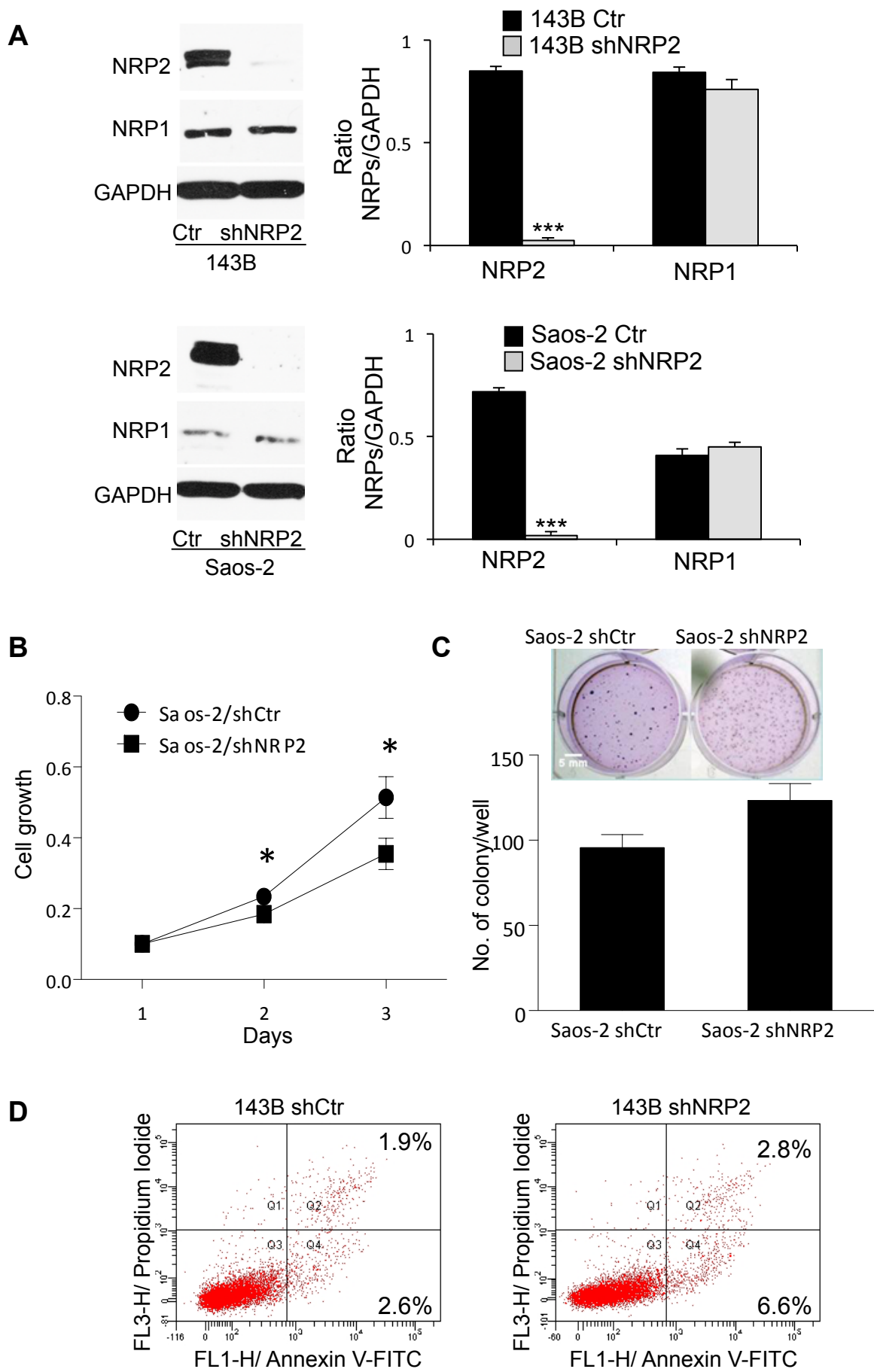

Suppl. Fig.2

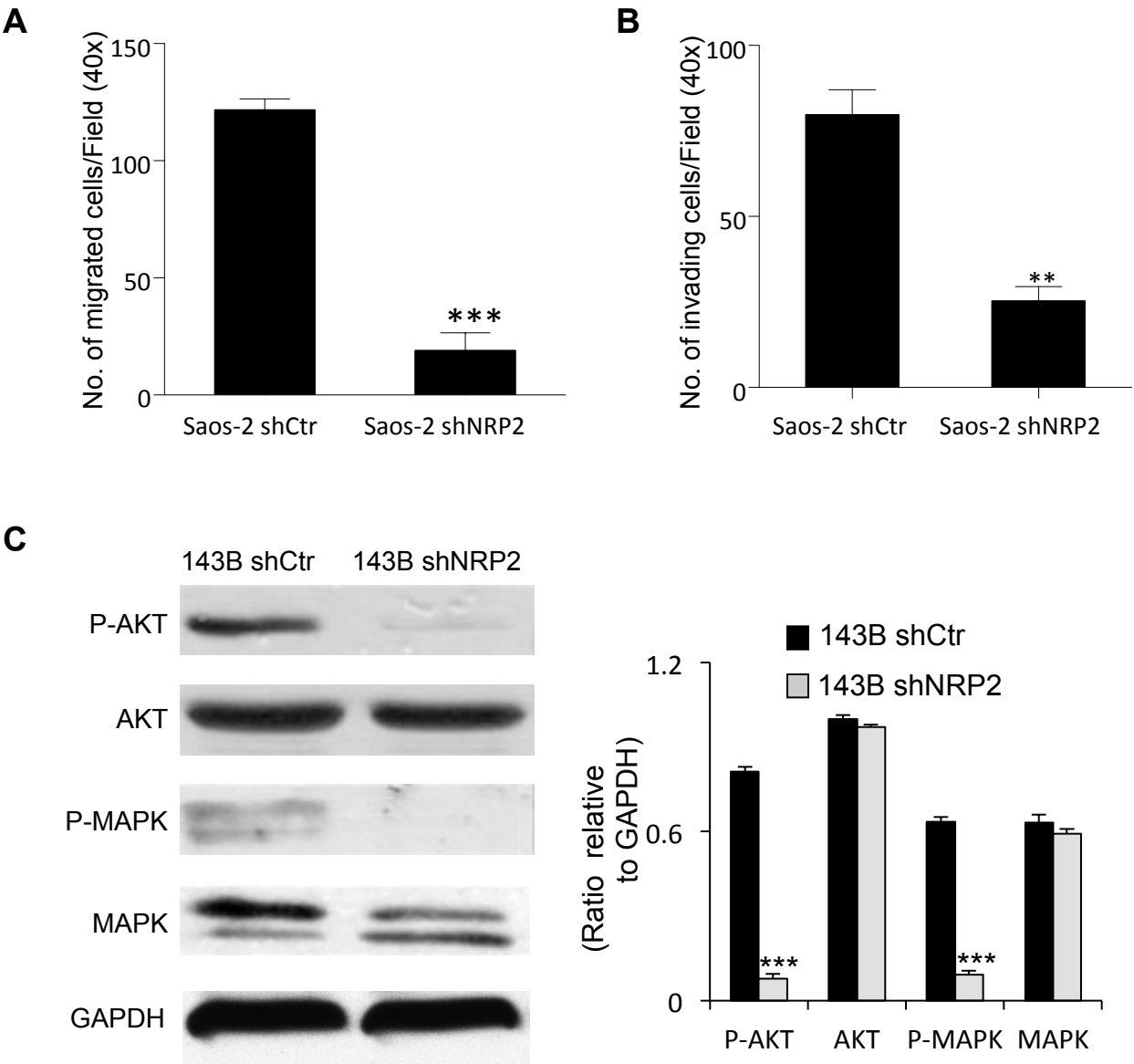

**Suppl. Fig.3**

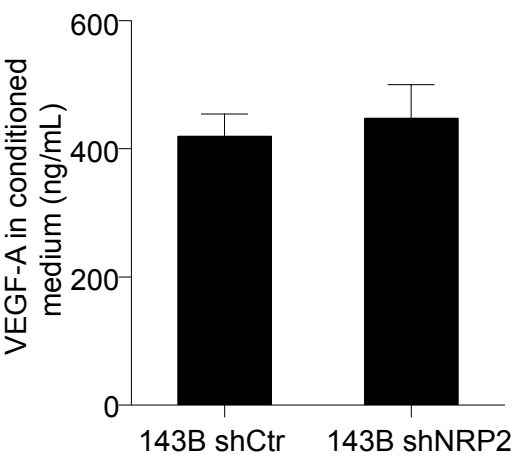

Supplement: Additional file 1: Figure S1. — NRP2 knockdown inhibited in vitro tumor growths. (A), NRP2 expression was specifically knocked down by NRP2 shRNA in 143B and Saos-2 cells, while NRP1 level was intact. Knockdown efficiency was determined by Western blotting and quantified by densitometric assay. (B), By MTT assay, NRP2 knockdown suppressed anchorage-dependent growth of Saos-2 cells. (C), Soft agar assay. NRP2 knockdown didn’t significantly reduce the number of colony formed by Saos-2 cells, but decreased the size of colony as shown in the representative images of soft agar (insert). (D) There were slightly higher percentage of early and late apoptosis in shNRP2 OS cells, compared to control cells. Figure S2. NRP2 depletion significantly suppressed migration and invasion of Saos-2 cells. (A), Migration assay. The BD chamber system without Matrigel coating was used to evaluate the migration of osteosarcoma Saos-2 cells transfected with shNRP2 and control vector. (B), Matrigel invasion assay was performed in BD chamber system coated with Matrigel, using shNRP2 and control vector transfected osteosarcoma Saos-2 cells. (C), Western blot and accompanying densitometry demonstrated the inhibition of AKT and MAPK phosphorylation by NRP2 knockdown in 143B cells. Figure S3. ELISA demonstrated no difference of VEGF-A level in the condition medium from shNRP2 and control vector transfected 143B cells. [file 12943_2015_359_MOESM1_ESM.pdf]
